# Perinatal Enterovirus Infection in Neonates: A Systematic Review

**DOI:** 10.1002/jmv.70362

**Published:** 2025-04-22

**Authors:** Cho Ryok Kang, Jung Hye Byeon, Hannah Cho, Juyoung Lee, Young June Choe

**Affiliations:** ^1^ Department of Nursing Wonkwang University Iksan Korea; ^2^ Department of Pediatrics Korea University College of Medicine and Korea University Anam Hospital Seoul Korea

**Keywords:** enterovirus infections, global health, infant, newborn, diseases, perinatal infections, pregnancy complications, infectious

## Abstract

Enteroviruses (EVs) are a common cause of neonatal infections, and perinatal EV infection can lead to severe neonatal disease, including sepsis‐like presentations, and adverse pregnancy outcomes. However, a comprehensive understanding of the prevalence and clinical manifestations of perinatal EV infection is lacking. This systematic review investigated the prevalence and clinical manifestations of perinatal EV infection. A comprehensive literature search was performed in PubMed, Embase, and KoreaMed up to August 26, 2024. Studies describing perinatal outcomes related to EV infection in neonates and pregnant women were included. Data extraction and quality assessment were performed independently by two reviewers. Nine studies (three each from America, Europe, and Asia) were included. Severe neonatal complications included sepsis‐like disease and death. Maternal symptoms included fever, uterine contractions, and rash. Perinatal EV infection prevalence ranged from 60% to 77.8% in severely affected neonates, 25% to 57.1% in infected pregnant women, and 4.6% to 46.1% in all infected newborns. Placental infection was confirmed in 38.3% of severe neonatal cases. This review highlights the global presence and potential severity of perinatal EV infections, underscoring the need for enhanced surveillance, standardized diagnostic protocols, and further research to inform effective prevention and management strategies.

## 1 Introduction

Perinatal enterovirus (EV) infections, encompassing maternal infections during pregnancy and subsequent transmission to the fetus or neonate, pose a significant yet often underestimated threat to maternal and infant health worldwide [1]. The clinical manifestations of these infections are diverse, ranging from mild, flu‐like symptoms in mothers to severe complications in neonates, including hepatitis, myocarditis, meningoencephalitis, and even fetal loss [2].

Despite their potential severity, the epidemiology of perinatal EV infections remains poorly understood, with considerable gaps in knowledge regarding incidence, prevalence, and risk factors across different geographical regions [3]. This scarcity of comprehensive data on incidence, prevalence, and clinical outcomes hinders effective prevention and management strategies, potentially leading to delayed diagnosis, inadequate treatment, and adverse maternal and neonatal outcomes.

To address this critical knowledge gap, we conducted a systematic review to synthesize existing evidence on perinatal EV infections from around the world. By providing a comprehensive overview of the current state of knowledge, including the prevalence, clinical manifestations, and maternal and neonatal outcomes associated with these infections, this review will contribute to a deeper understanding of the global burden of perinatal EV infections.

## 2 Methods

### 2.1 Search Strategy and Data Sources

A literature search was conducted in PubMed, Embase, and KoreaMed by a trained medical librarian (Ms. Eun‐Ji Kang) from inception up to 26 August 2024. We utilized institutional subscriptions available through the Korea University Medical Library to access full‐text articles across multiple databases, including PubMed, Embase, and KoreaMed. We acknowledge that full‐text availability may vary significantly across different institutions; therefore, studies for which full texts were not accessible through these subscriptions were excluded. The search strategy initially developed for MEDLINE using keywords and MeSH terms was applied to other databases. The exact search strategies are outlined in Table S1. There were no language restrictions. Reference lists of included articles and relevant literature identified through manual searches were also screened for additional publications. The study protocol has been registered and published with PROSPERO (CRD42024603509). This study was exempt from Institutional Review Board approval as a systematic review.

### 2.2 Study Selection

The titles and abstracts of the records identified were independently screened by two reviewers (C.R.K., J.H.B.). The same reviewers for all identified articles conducted a full‐text review. Inclusion criteria for selecting articles include studies that describe perinatal outcomes related to EV infection in neonates and pregnant women in different countries and clinical cohorts, including surveillance reports or original papers of observational studies including cross‐sectional studies, case‐control studies, prospective, and retrospective studies (cohorts, case‐control studies, and registries). Exclusion criteria were (a) duplicate studies; (b) systematic review ± meta‐analysis; (c) nonoriginal studies including review, comments, editorials, case reports, guidelines, and book chapters; (d) intervention studies (randomized and clinical controlled trials); (e) studies on comparison or validation of detection methods; (f) no data on the perinatal EV infection in neonates; and (g) no access to full‐text.

### 2.3 Data Extraction and Quality Assessment

Two reviewers (C.R.K., Y.J.C.) independently extracted data on study details, such as first author, publication year, country, study period, study design, study setting, study population characteristics, perinatal EV incident cases, symptoms of perinatal EV cases, sample type, and EV genotypes. Discrepancies were resolved through discussion. The quality of all studies that met the inclusion criteria was assessed using the Newcastle‐Ottawa Scale. Studies scoring more than 7 points were considered to be of good quality, those scoring 5–7 points were considered to be of moderate quality, and those scoring less than 5 points were considered to be of poor quality [4].

## 3 Results

### 3.1 Study Selection

**Figure 1:**
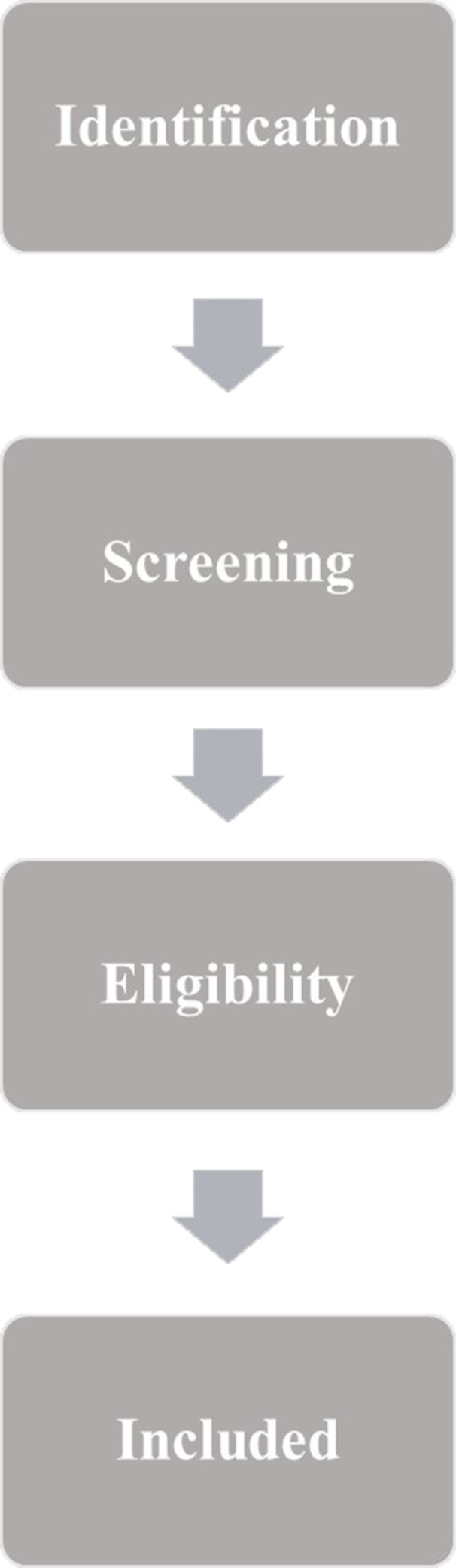
PRISMA flow diagram.

Figure 1 illustrates the study selection process using a PRISMA flow diagram, detailing the steps from initial identification through screening and full‐text review to the final inclusion of studies. The initial search yielded 1810 potential references in a systematic search of sources. A total of 372 articles were duplicated, and 1338 were excluded after screening the titles and abstracts of the articles. After reviewing full‐text articles, 91 articles were excluded. Finally, nine studies were included in the systematic review. The studies were at moderate risk of bias (Tables S2 and S3).

### 3.2 Study Characteristics

Table 1 lists the major characteristics of the included studies. The included studies originated from America (*n* = 3; United States) [5, 6, 7], Europe (*n* = 3; France, Italy) [8, 10, 12], and Asia (*n* = 3; China, Taiwan, and Israel) [9, 11, 13]. There were nine studies, five of which were retrospective [6, 9, 10, 11, 13], and one was a case‐control study [6]. The studies were hospital‐based, and the majority were single‐center studies [5, 6, 9, 10, 11], including two studies reported from neonatal intensive care units (NICUs) [9, 10]. Regarding the study population, six studies included neonates with EV infection [7, 9, 10, 11, 12, 13], two studies included pregnant women [5, 8], and one study reported placental tissue findings [6]. In five studies, laboratory tests for EV infection were conducted on neonates and their mothers to identify perinatal outcomes [5, 8, 11, 12, 13]. Other studies examined perinatal infection based on the symptom onset of EV‐infected newborns [9] or whether the mother had related clinical symptoms [7, 10]. Regarding detection methods for diagnosing EVs, virus isolation was conducted in two studies [5, 9], and molecular assay based on reverse transcription‐polymerase chain reaction (RT‐PCR) was conducted in seven studies [6, 7, 8, 10, 11, 12, 13].

**Table 1:** Summary of the characteristics of studies included.

First author	Publication year	Country	Study period	Study design	Study setting	Study population characteristics	Perinatal EV prevalence rate (%)	Neonates of perinatal EV infections				Pregnant women with perinatal EV infections		
								Symptoms	Sample type	Detection method	EV genotype(s)	Symptoms	Sample type	Detection method/EV genotype(s)
Modlin [5]	1981	United States	1979 (September 13 to October 1)	Prospective	Boston Hospital for Women	7 virus‐positive mothers among 194 consecutive women admitted to the labor floor	57.1% (4/7)	Nondetectable illness during the 2 weeks of observation	Throat, rectum	Viral isolation	E11 (4)	Only 1 virus‐positive woman reported a brief, afebrile respiratory illness with a productive cough that had occurred approximately 2 weeks before delivery	Throat, rectum	Viral isolation
														—
														E11 (4)
Satosar [6]	2004	United States	NA	Retrospective	Ohio State University Medical Center Pathology archives (placental tissue fixed in 10% buffered formalin and embedded in paraffin)	60 placental tissues from fetal or neonatal death (11 cases) or idiopathic severe respiratory distress or central nervous system‐related symptoms at birth (49 cases)	38.3% (23/60)	Low APGAR score (≤ 6 for 1 and 5 min), respiratory distress (requiring assisted ventilation at birth), neurologic sequela (either seizures in the neonatal period or developmental delays after 1 year), death of unknown etiology	Placentas were taken from areas where there were no gross abnormalities	RT‐PCR	Enterovirus (1), coxsackie virus types A (6) and B (16)	NA	NA	NA
Wikswo [7]	2009	United States	2007–2008	Prospective	20 states notified the CDC directly	6 neonatal deaths due to CVB1 infection	66.6% (4/6)	Multisystem disease within the first 4 days of life. 3 neonates had both myocarditis and hepatic involvement.	Virus isolates or original specimens	RT‐PCR	CVB1 (4)	Febrile illness or chorioamnionitis around the time of delivery	NA	NA
Khediri [8]	2018	France	2016 (February–June)	Prospective	3 centers in the Paris region	4 enterovirus infections in the population of 31 febrile pregnant women	25% (1/4)	Viral enterovirus meningitis, severe cardiogenic shock following myocarditis with left ventricular dysfunction, multivisceral failure	Blood	RT‐PCR	Enterovirus (1)	Fever (39°C), uterine contractions, nonpruritic maculopapular	Blood	RT‐PCR
														/
														Enterovirus (1)
Ho [9]	2020	Taiwan	2018 (May–June)	Retrospective	NICUs in Chang Gung Memorial Hospital	6 neonates among E11 confirmed 10 patients in the NICU outbreak	33.3% (2/6)	Fever, oral ulcer, petechiae, hepatitis, coagulopathy, thrombocytopenia, tachycardia	CSF, urine, throat, rectum	Viral isolation	E11	NA	NA	NA
							※2 cases were classified as vertical transmissions (disease onset on Days 1 and 7 of life)							
Bersani [10]	2020	Italy	2004–2018	Retrospective	NICU at the Bambino Gesù Children's Hospital in Rome	5 cases infected with enterovirus among 10 cases of neonatal ALF (all were late preterm infants [32–36 weeks])	60% (3/5)	Neonatal acute liver failure, 1 infant died as a result of ALF	CSF, blood, bone marrow	RT‐PCR	CVB3 (1), CVB5 (2)	Fever and mild diarrhea 2 months before delivery, 1/125 anti‐coxsackie B5 neutralizing antibodies titer	NA	NA
							※2 dizygotic twin siblings							
Belov [11]	2021	Israel	2014–2019	Retrospective	A tertiary university‐affiliated medical center	13 neonates who had been diagnosed with confirmed peripartum enterovirus infection, defined as an infection occurring within 2 weeks of birth	46.1% (6/13)	Sepsis, meningitis, DIC, pulmonary hemorrhage, hepatitis, IVH, 3 of 6 neonates died	CSF, stool	RT‐PCR	Enterovirus (4), CVB3 (2)	Fever, abdominal pain, diarrhea, cough, sore throat, arthralgia, rhinorrhea, muscle pain	Stool	RT‐PCR
							※6 neonates were born to 5 mothers							/
														Enterovirus (4), CVB5 (1)
Grapin [12]	2023	France	July 2022 to April 2023	Prospective	The hospital‐based EV surveillance (covering approximately 100 hospitals distributed in all French regions)	9 severe neonatal infections caused by a new variant of echovirus 11 (8 were from twin pregnancies)	77.8% (7/9)	Septic shock requiring active resuscitation, acute liver failure, multivisceral failure, 5 of 7 neonates died	CSF, stool, plasma, throat (liver biopsy, psoas biopsy)	RT‐PCR	E11 (7)	Gastrointestinal symptoms or fever during the 3 days before or at delivery	Blood	RT‐PCR
							※7 neonates were born to 4 mothers							/
														E11 (4)
Yang [13]	2024	China	2019–2022	Retrospective	34 hospitals in 12 cities across southern China	260 newborns aged 0–28 days with EV infection	4.6% (12/260)	Sepsis‐like disease, meningitis, myocarditis, HFMD, or other symptoms compatible with EV infection	Stool	RT‐PCR	E11 (4), E6 (4), CVB2 (2), CVA9 (1), E19 (1)	NA	Stool	RT‐PCR
														/
														E11 (4), E6 (4), CVB2 (2), CVA9 (1), E19 (1)

### 3.3 Perinatal Outcomes and EV Incident Cases

Regarding the perinatal outcomes of EV‐PCR‐positive in newborn–mother pairs, severe complications including sepsis‐like disease or death occurred in newborns [8, 11, 12, 13], and in mothers, fever was the main symptom, and other symptoms included uterine contractions and maculopapular [8], gastrointestinal or respiratory symptoms (Table 1) [11, 12]. In instances where severe neonatal infection was inferred from either neonatal symptoms or maternal clinical history, we included studies reporting neonatal disease onset on Days 1 and 7 of life [9], maternal febrile illness or chorioamnionitis around delivery [7], as well as cases with maternal fever accompanied by mild diarrhea 2 months before delivery or a neutralizing antibody titer of 1/125 for coxsackie B5 [10] were included.

The reported incident cases of EV infection varied considerably across the included studies. In newborns presenting with severe complications—such as sepsis‐like disease or death—the incident case proportion of severe infection, calculated as the number of neonates with severe outcomes divided by the total number of neonates presenting with severe clinical symptoms, ranged from 60% to 77.8% (Table 1) [7, 10, 12]. Among pregnant women with laboratory‐confirmed EV infection, 25%–57.1% of incident cases were symptomatic, based on the number of women exhibiting clinical symptoms relative to the total number with confirmed EV infection [5, 8]. Furthermore, when considering all EV‐infected newborns, regardless of disease severity, the proportion of severe infections ranged from 4.6% to 46.1% of incident cases [9, 11, 13]. In addition, one study evaluating placental tissues from newborns with severe clinical courses identified EV infection in 38.3% of incident cases examined [6].

## 4 Discussion

This systematic review identified and analyzed nine studies on perinatal EV infections, providing valuable insights into their global prevalence, clinical manifestations, and maternal and neonatal outcomes. Neonatal EV infections can be categorized into perinatal cases and those acquired postnatally, distinguished by timing and maternal involvement [14]. Perinatal EV infections typically manifest within the first week of life and often coincide with maternal peripartum illness, whereas neonatal infections acquired from family or nosocomial exposure usually present later without any maternal symptoms. Clinically, both groups present with a similar sepsis‐like syndrome in the neonates, which can progress to severe complications such as hepatic necrosis with coagulopathy, meningoencephalitis, or myocarditis. However, vertically infected infants are at higher risk of fulminant multiorgan disease, likely because they lack passively transferred maternal antibodies, whereas later‐onset cases may be partially mitigated by the presence of maternal antibodies if the mother has prior immunity [15]. Our findings highlight the significant threat these infections pose to maternal and child health worldwide, underscoring the urgent need for enhanced surveillance, standardized diagnostic protocols, and further research to inform effective prevention and management strategies.

It is important to recognize the diversity of EVs and their associated clinical manifestations. As highlighted in the previous studies, there are 70 distinct enteroviral serotypes, each capable of causing a range of illnesses, from mild fever and rashes to severe conditions like myocarditis and central nervous system disease [16]. This diversity underscores the importance of identifying the specific EV serotypes associated with perinatal infections and their respective contributions to maternal and neonatal complications. Further research focusing on the prevalence and clinical impact of different EV serotypes in perinatal infections will be crucial in developing targeted prevention and management strategies.

The included studies revealed that perinatal EV infections can lead to severe complications in both mothers and neonates. In mothers, infections were associated with fever, uterine contractions, maculopapular rash, gastrointestinal, and respiratory symptoms [5, 8, 11, 12]. In neonates, infections resulted in severe outcomes, including sepsis‐like disease, multiorgan failure, and even death [3, 6, 7, 10, 13]. These findings emphasize the potential severity of these infections and the importance of early detection and appropriate management. When investigating sepsis‐like features in a newborn, it is crucial to consider the maternal history and potential exposure to EVs during pregnancy [17]. To further enhance our understanding and management of perinatal EV infections, it is essential to collect more cases and conduct further research. This will help identify specific maternal risk factors associated with severe neonatal outcomes, potentially leading to the development of preventive or management strategies.

The heterogeneity in the clinical presentation of perinatal EV infections is evident, with maternal symptoms often being mild and nonspecific, such as fever, mild gastrointestinal or respiratory complaints, and transient rashes, while neonates may develop severe conditions including sepsis, meningitis, myocarditis, and acute liver failure [6, 10]. The difference in disease severity between mothers and neonates suggests that maternal infections may not predict adverse outcomes, whereas neonates remain highly vulnerable due to their immature immune responses. The timing of infection is also critical; neonates exposed to maternal infection near delivery, as indicated by fever or chorioamnionitis, tend to show serious symptoms within the first few days of life, while earlier or subclinical maternal infections may lead to less severe disease in neonates [5, 8]. Moreover, variability in EV serotypes, with echovirus 11 and coxsackie B viruses frequently associated with severe neonatal disease, further contributes to these differences [5, 7]. These findings underscore the need for enhanced surveillance with detailed maternal histories, early diagnostic testing, and tailored intervention strategies for this vulnerable population.

The limitations of this systematic review include the heterogeneity of included studies, the potential for publication bias, and the reliance on observational data. The small number of studies included and the variability in their methodologies limit the generalizability of our findings. Publication bias may have led to the overrepresentation of studies with positive or significant findings. Additionally, the reliance on observational studies, with their inherent risk of bias, may affect the strength of evidence for causal relationships between perinatal EV infections and adverse maternal and neonatal outcomes. Despite these limitations, this systematic review provides a comprehensive overview of the current evidence on perinatal EV infections. Our findings underscore the need for further research to address the knowledge gaps identified, including the development of standardized diagnostic protocols and surveillance systems to enable more accurate and comparable estimates of the global burden of these infections.

In conclusion, this systematic review highlights the global presence and potential severity of perinatal EV infections, emphasizing the diverse range of maternal and neonatal complications. Our findings underscore the critical need for healthcare providers to maintain a high index of suspicion for EV infection when encountering neonates with sepsis‐like features or other severe clinical presentations.

## Author Contributions

Cho Ryok Kang and Jung Hye Byeon contributed equally to the study conception, design, and manuscript drafting. Hannah Cho, Juyoung Lee, and Young June Choe participated in data extraction, analysis, and critical revision of the manuscript. All authors have read and approved the final version of the manuscript.

## Conflicts of Interest

The authors declare no conflicts of interest.

## Supporting information

EV SR supplementary.

## Data Availability

All data generated or analyzed during this study are included in this published article and its supplementary information files. The data sets utilized in this systematic review are publicly available from the databases referenced (PubMed, Embase, and KoreaMed). Additional details or data can be obtained from the corresponding author upon reasonable request.

## References

[1] C. Tomatis Souverbielle , G. Erdem , and P. J. Sánchez , “Update on Nonpolio Enterovirus and Parechovirus Infections in Neonates and Young Infants,” Current Opinion in Pediatrics 35 (2023): 380–389. 36876331 10.1097/MOP.0000000000001236

[2] N. Harik and R. L. DeBiasi , “Neonatal Nonpolio Enterovirus and Parechovirus Infections,” Seminars in Perinatology 42 (2018): 191–197. 29526382 10.1053/j.semperi.2018.02.007

[3] J. A. Jenista , K. R. Powell , and M. A. Menegus , “Epidemiology of Neonatal Enterovirus Infection,” Journal of Pediatrics 104 (1984): 685–690. 6325656 10.1016/s0022-3476(84)80944-0

[4] G. A. Wells , B. Shea , D. O'Connell , et al., The Newcastle‐Ottawa Scale (NOS) for Assessing the Quality of Nonrandomised Studies in Meta‐Analyses (The Ottawa Hospital, 2011), http://www.ohri.ca/programs/clinical_epidemiology/oxford.asp.

[5] J. F. Modlin , B. F. Polk , P. Horton , P. Etkind , E. Crane , and A. Spiliotes , “Perinatal Echovirus Infection: Risk of Transmission During a Community Outbreak,” New England Journal of Medicine 305 (1981): 368–371. 7254269 10.1056/NEJM198108133050703

[6] A. Satosar , N. C. Ramirez , D. Bartholomew , J. Davis , and G. J. Nuovo , “Histologic Correlates of Viral and Bacterial Infection of the Placenta Associated With Severe Morbidity and Mortality in the Newborn,” Human Pathology 35 (2004): 536–545. 15138926 10.1016/j.humpath.2004.01.015

[7] M. E. Wikswo , N. Khetsuriani , A. L. Fowlkes , et al., “Increased Activity of Coxsackievirus B1 Strains Associated With Severe Disease Among Young Infants in the United States, 2007–2008,” Clinical Infectious Diseases 49 (2009): e44–e51. 19622041 10.1086/605090

[8] Z. Khediri , C. Vauloup‐Fellous , A. Benachi , J. M. Ayoubi , L. Mandelbrot , and O. Picone , “Adverse Effects of Maternal Enterovirus Infection on the Pregnancy Outcome: A Prospective and Retrospective Pilot Study,” Virology Journal 15 (2018): 70. 29661198 10.1186/s12985-018-0978-7 PMC5902830

[9] S. Y. Ho , C. H. Chiu , Y. C. Huang , et al., “Investigation and Successful Control of an Echovirus 11 Outbreak in Neonatal Intensive Care Units,” Pediatrics & Neonatology 61 (2020): 180–187. 31669107 10.1016/j.pedneo.2019.09.012

[10] I. Bersani , C. Auriti , F. Piersigilli , et al., “Neonatal Acute Liver Failure due to Enteroviruses: A 14 Years Single NICU Experience,” Journal of Maternal–Fetal & Neonatal Medicine 33 (2020): 2576–2580. 30513031 10.1080/14767058.2018.1555806

[11] Y. Belov , A. Many , I. Givon , et al., “Maternal Presentation and Neonatal Outcome in Peripartum Enterovirus Infection,” Acta Paediatrica 110 (2021): 1483–1489. 33251624 10.1111/apa.15703

[12] M. Grapin , A. Mirand , D. Pinquier , et al., “Severe and Fatal Neonatal Infections Linked to a New Variant of Echovirus 11, France, July 2022 to April 2023,” Eurosurveillance 28 (2023): 2300253. 37261730 10.2807/1560-7917.ES.2023.28.22.2300253 PMC10236930

[13] X. Yang , Y. Wu , H. Zhao , P. Liu , L. Liang , and A. Yin , “Emergence and Circulation of Enterovirus B Species in Infants in Southern China: A Multicenter Retrospective Analysis,” Virulence 15 (2024): 2329569. 38555521 10.1080/21505594.2024.2329569 PMC10984118

[14] Y. Y. Chuang and Y. C. Huang , “Enteroviral Infection in Neonates,” Journal of Microbiology, Immunology and Infection 52 (2019): 851–857. 10.1016/j.jmii.2019.08.018 31607572

[15] M. Zhang , H. Wang , J. Tang , et al., “Clinical Characteristics of Severe Neonatal Enterovirus Infection: A Systematic Review,” BMC Pediatrics 21 (2021): 127. 33722228 10.1186/s12887-021-02599-y PMC7958388

[16] M. H. Sawyer , “Enterovirus Infections: Diagnosis and Treatment,” Seminars in Pediatric Infectious Diseases 13 (2002): 40–47. 12118843 10.1053/spid.2002.29756

[17] I. Bellos , A. Pandita , and R. Panza , “Maternal and Perinatal Outcomes in Pregnant Women Infected by SARS‐CoV‐2: A Meta‐Analysis,” European Journal of Obstetrics & Gynecology and Reproductive Biology 256 (2021): 194–204. 33246205 10.1016/j.ejogrb.2020.11.038 PMC7664337

