# Supplementary material for: Perinatal Enterovirus Infection in Neonates: A Systematic Review
Source: J Med Virol. 2025 Apr 22;97(5):e70362. doi: 10.1002/jmv.70362 (PMC12015150; doi:10.1002/jmv.70362)
Supplement: Supplementary file 1 — EV SR supplementary. [file JMV-97-e70362-s001.docx]

**Supplementary online**

Tables

**Table S1.** Search strategy

**Table S2-3.** Risk of bias for included studies

**Table S1.** Search strategy

| **Search strategy in PubMed** | |
| --- | --- |
| Steps | Searches |
| #1 | Enterovirus[mh] OR Enterovirus*[tw] OR coxsackie*[tw] OR coxsachie*[tw] OR entero virus*[tw] OR echovirus*[tw] OR ECHO virus*[tw] OR NPEV[tw] |
| #2 | Enterovirus Infections[mh:noexp] OR Coxsackievirus Infections[mh] OR Echovirus Infections[mh] OR enteroviral infect*[tw] OR herpangina*[tw] OR HFMD[tw] OR hand foot and mouth disease*[tw] OR hand foot mouth disease*[tw] |
| #3 | #1 OR #2 |
| #4 | Infant, Newborn[mh] OR Infant, Newborn, Diseases[mh:noexp] OR newborn*[tw] OR neonate*[tw] OR neonatal[tw] OR perinatal period[tw] |
| #5 | Incidence[mh] OR Prevalence[mh] OR incidence*[tw] OR prevalence*[tw] OR epidemiol*[tw] OR proportion*[tw] OR surveillance*[tw] |
| #6 | #3 AND #4 AND #5 |
| #7 | (Animals[mh] NOT Humans[mh]) OR Models, Animal[mh:noexp] OR Disease Models, Animal[mh] OR Animal Experimentation[mh] |
| #8 | Review[pt] OR Systematic Reviews as Topic[mh] OR Systematic Review[pt] OR Meta-Analysis as Topic[mh] OR Meta-Analysis[pt] OR systematic review*[tw] OR systematic overview*[tw] OR meta-analys*[tw] OR metaanalys*[tw] OR scoping review*[tw] OR scoping literature review*[tw] OR systematic literature review*[tw] OR umbrella review*[tw] OR meta-regression[tw] OR review*[ti] |
| #9 | Case Reports[pt] OR case report*[ti] OR case stud*[ti] |
| #10 | #6 NOT #7 NOT #8 NOT #9 |
| **Search strategy in Embase** | |
| #1 | Enterovirus/exp OR (Enterovirus* OR coxsackie* OR coxsachie* OR echovirus* OR NPEV):ti,ab,kw OR ((entero OR ECHO) NEXT/1 virus*):ti,ab,kw |
| #2 | ('Enterovirus infection'/de OR 'Coxsackie virus infection'/de OR 'Echovirus infection'/de OR herpangina/de OR 'hand foot and mouth disease'/de OR ('enteroviral infect*' OR herpangina* OR HFMD):ti,ab,kw OR (hand NEXT/2 foot NEXT/2 mouth NEXT/2 disease*):ti,ab,kw) |
| #3 | #1 OR #2 |
| #4 | newborn/de OR 'newborn disease'/de OR 'perinatal period'/de OR (newborn* OR neonate* OR neonatal OR 'perinatal period'):ti,ab,kw |
| #5 | incidence/de OR prevalence/de OR 'epidemiological surveillance'/exp OR (incidence* OR prevalence* OR epidemiol* OR proportion* OR surveillance*):ti,ab,kw |
| #6 | #3 AND #4 AND #5 |
| #7 | (animal/exp NOT human/exp) OR 'animal model'/exp OR 'animal experiment'/exp OR [animal cell]/lim OR [animal experiment]/lim OR [animal model]/lim OR [animal tissue]/lim |
| #8 | review/exp OR review:it OR [review]/lim OR 'systematic review'/de OR 'systematic review (topic)'/de OR [cochrane review]/lim OR [systematic review]/lim OR 'meta analysis'/exp OR 'meta analysis (topic)'/de OR [meta analysis]/lim OR ((systematic OR scoping OR umbrella) NEXT/2 (review* OR overview*)):ti,ab,kw OR (meta-analys* OR meta-regression):ti,ab,kw OR review*:ti |
| #9 | ('case report'/de OR 'conference abstract'/it OR 'conference paper'/it OR 'conference review'/it OR (case NEXT/1 (report* OR stud*)):ti) |
| #10 | #6 NOT #7 NOT #8 NOT #9 |
| **Search strategy in KoreaMed** | |
| #1 | Enterovirus* OR coxsackie* OR coxsachie* OR "entero virus" OR echovirus* OR "ECHO virus" OR NPEV OR "enteroviral infection" OR herpangina* OR HFMD OR "hand foot and mouth disease" OR "hand foot mouth disease" |
| #2 | newborn* OR neonate* OR neonatal OR "perinatal period" |
| #3 | incidence* OR prevalence* OR epidemiol* OR proportion* OR surveillance* |
| #4 | #1 AND #2 AND #3 |

*Coxsackie viruses, a subgroup of enteroviruses, are frequently implicated in perinatal infections and can cause a spectrum of clinical manifestations ranging from mild febrile illness to severe neonatal complications.

# Table S2. Risk of bias for cohort studies included in the systematic review

|  | Selection | | | | Comparability | | Exposure | | | Total score |
| --- | --- | --- | --- | --- | --- | --- | --- | --- | --- | --- |
|  | **Representativeness of the exposed cohort** | **Selection of the non-exposed cohort** | **Ascertainment of exposure** | **Outcome of interest not present at start** | **Comparability: age and sex** | **Comparability: additional factors** | **Ascertainment of outcome** | **Follow-up long enough** | **Adequacy of follow-up** |  |
| Modlin 1981 | 0 | 0 | 1 | 1 | 0 | 0 | 1 | 1 | 1 | 5 |
| Khediri 2018 | 0 | 0 | 1 | 1 | 0 | 0 | 1 | 1 | 1 | 5 |
| Belov 2021 | 0 | 0 | 1 | 1 | 0 | 0 | 1 | 1 | 1 | 5 |
| Grapin 2023 | 1 | 0 | 1 | 1 | 0 | 0 | 1 | 1 | 1 | 6 |
| Yang 2024 | 0 | 0 | 1 | 1 | 0 | 0 | 1 | 1 | 1 | 5 |
| Wikswo 2009 | 1 | 0 | 1 | 1 | 0 | 0 | 1 | 1 | 1 | 6 |
| Ho 2020 | 0 | 0 | 1 | 1 | 0 | 0 | 1 | 1 | 1 | 5 |
| Bersani 2020 | 0 | 0 | 1 | 1 | 0 | 0 | 1 | 1 | 1 | 5 |

# Table S3. Risk of bias for case-control study included in the systematic review

|  | Selection | | | | Comparability | | Exposure | | | Total score |
| --- | --- | --- | --- | --- | --- | --- | --- | --- | --- | --- |
|  | **Case definition adequate** | **Representativeness of cases** | **Selection of controls** | **Definition of controls** | **Comparability: age and sex** | **Comparability: additional factors** | **Ascertainment of exposure** | **Case and controls: same ascertainment method** | **Case and controls: same nonresponse rate** |  |
| Satosar 2004 | 1 | 0 | 0 | 1 | 0 | 1 | 1 | 1 | 0 | 5 |
